# Collaborative SAR Modeling and Prospective In Vitro Validation of Oxidative Stress Activation in Human HepG2 Cells

**DOI:** 10.1021/acs.jcim.3c00220

**Published:** 2023-08-24

**Authors:** Olivier J. M. Béquignon, Jose C. Gómez-Tamayo, Eelke B. Lenselink, Steven Wink, Steven Hiemstra, Chi Chung Lam, Domenico Gadaleta, Alessandra Roncaglioni, Ulf Norinder, Bob van de Water, Manuel Pastor, Gerard J. P. van Westen

**Affiliations:** † Leiden Academic Centre for Drug Research, Leiden University, Wassenaarseweg 76, 2333 AL Leiden, The Netherlands; ‡ Research Programme on Biomedical Informatics (GRIB), Department of Medicine and Life Sciences, Hospital del Mar Medical Research Institute, Universitat Pompeu Fabra, Carrer del Dr. Aiguader 88, 08002 Barcelona, Spain; § Laboratory of Environmental Chemistry and Toxicology, Department of Environmental Health Sciences, IRCCS—Istituto di Ricerche Farmacologiche Mario Negri, Via la Masa 19, 20156 Milano, Italy; ∥ MTM Research Centre, School of Science and Technology, Örebro University, SE-70182 Örebro, Sweden

## Abstract

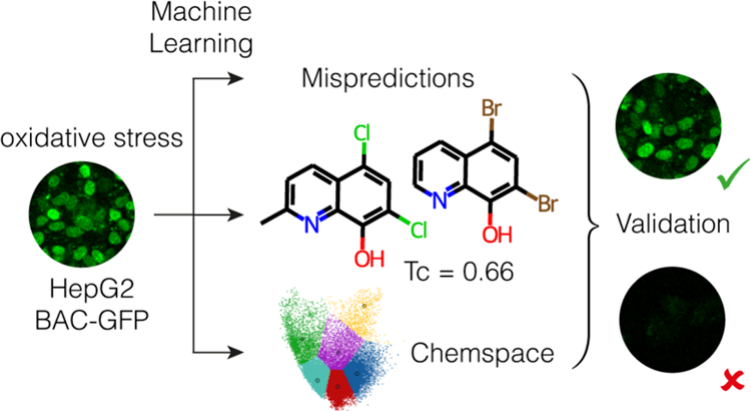

Oxidative stress is the consequence of an abnormal increase of reactive oxygen species (ROS). ROS are generated mainly during the metabolism in both normal and pathological conditions as well as from exposure to xenobiotics. Xenobiotics can, on the one hand, disrupt molecular machinery involved in redox processes and, on the other hand, reduce the effectiveness of the antioxidant activity. Such dysregulation may lead to oxidative damage when combined with oxidative stress overpassing the cell capacity to detoxify ROS. In this work, a green fluorescent protein (GFP)-tagged nuclear factor erythroid 2-related factor 2 (NRF2)-regulated sulfiredoxin reporter (Srxn1-GFP) was used to measure the antioxidant response of HepG2 cells to a large series of drug and drug-like compounds (2230 compounds). These compounds were then classified as positive or negative depending on cellular response and distributed among different modeling groups to establish structure–activity relationship (SAR) models. A selection of models was used to prospectively predict oxidative stress induced by a new set of compounds subsequently experimentally tested to validate the model predictions. Altogether, this exercise exemplifies the different challenges of developing SAR models of a phenotypic cellular readout, model combination, chemical space selection, and results interpretation.

## Introduction

In silico models can be used as a cheap and fast tool to estimate toxicity in the early stages of drug discovery and can be applied to any compound based on the chemical structure, whether the molecule has been already synthesized or not.^1−3^ In general, in silico toxicology methods can be classified as statistical-based or knowledge-based approaches.^1,4,5^ Knowledge-based models rely on previously acquired knowledge of a toxicological phenomenon, for instance, by flagging structural alerts in molecules that were previously identified to be toxic by experts, while statistical-based models identify relationships between descriptors of molecules and their phenotypical endpoint. The resulting statistical model is then able to correlate and find associations between biological properties and structure, but investigating a causative link typically requires further research.

Herein, the authors present a complete modeling exercise from the experimental testing of a bioactive compound library to the application of machine learning models on new compounds and their further selection for a prospective experiment and analysis. As a toxicological target for this investigation, oxidative stress was chosen as a central event for many adverse outcome pathways (AOP) due to its relationship with different toxicity mechanisms and diseases such as Parkinson’s,^6^ cancer, chronic fatigue,^7^ and drug-induced liver injury (DILI). The Spectrum Collection compound library (Microsource Discovery Systems) was selected as the training dataset because of its wide representation of bioactive compounds, providing an extensive chemical space coverage of the drug-like compounds.

Under normal physiological conditions, reactive oxygen species (ROS) are generated from internal metabolism and external exposure at levels that the cell detoxification machinery can handle.^8−10^ When there is an imbalance of ROS, tyrosine kinases dissociate from the nuclear factor erythroid 2-related factor (NRF2). This activates a response against oxidative stress by expressing antioxidant enzymes related to antioxidant species synthesis, such as glutathione (reduced nicotinamide adenine dinucleotide phosphate (NADPH)), quinone oxidoreductase, and heme oxygenase-1.^9^ When dysregulation occurs and the antioxidant system is not able to keep redox homeostasis, cells suffer oxidative damage through lipid peroxidation and protein and DNA oxidation. Uncontrolled oxidative damage can lead to cell death by apoptotic signaling or to activation of the inflammasome assembly.^11,12^ Time-sustained excess of ROS is related to cancer, chronic diseases, and toxicity.^13,14^ In the mitochondria, ROS are generated in higher quantities, and hence the ROS scavenging systems show higher expression.^14^ Xenobiotics can produce a disruption in ROS homeostasis in different ways: metabolic processes can generate ROS directly, but they can also disrupt physiological processes involved in redox reactions, like the mitochondrial electron transportation chain, or affect the expression of genes involved in ROS detoxification. The metabolism of xenobiotics takes place mostly in the liver, making it a hotspot for oxidative stress study. In the present study, the experimental characterization of the potential ROS disruption of xenobiotics was carried out using a HepG2 reporter cell line^15^ containing the genomic modified sulfiredoxin-green fluorescent protein (Srxn1-GFP) fusion protein exposed to 30 nM bardoxolone methyl (methyl-2-cyano 3,12-dioxooleano-1,9-dien-28-oate) (CDDO-Me) as the NRF2 pathway activator, enabling a dynamic range for up- and downregulation for compounds tested with respect to the CDDO-Me baseline activation.^16^ This HepG2 SRXN1-GFP reporter cell line’s response to DILI-related compounds had previously been shown to strongly correlate to primary human hepatocytes (PHH) not only in terms of directionality of gene expression but also the relationships between these genes reflecting pathway regulation.^17−19^

Predicting if a compound produces oxidative stress is challenging. Although some substructures are known to be prone to form ROS metabolites, like quinoid-containing compounds,^20^ these alerts only account for ROS generation from xenobiotics. Thus, such substructure filters would neglect other mechanisms related to, for instance, the inhibition of proteins involved in the antioxidant cell machinery. There are many sources of oxidative stress, and the underlying mechanisms are not totally characterized, preventing from the definition of a comprehensive definition of structural alerts. We hypothesize that statistical models trained using large datasets have the potential to overcome these limitations of rule-based systems. Other works have also reported high predictive performance, though both quantity and quality of the data are key factors.^21^ Thanks to the advances in both data quality, quantity, and machine learning techniques, statistical-based models have regained interest.^22−24^ However, modeling oxidative stress as a single endpoint using cheminformatics approaches has not been addressed so far, probably due to the different nature of its underlying mechanisms and the complex and not well-understood translation to diseases.

In this work, we explore the ability of in silico statistical models to predict oxidative stress, analyzing models created by different research groups working on the EU-ToxRisk project.^25^

Three-dimensional (3D) molecular descriptors were not considered in this study as (i) they previously showed limited improvement or deterioration of the model performance with endpoints related to hepatotoxicity, (ii) the molecular structures considered herein had limited dependence on 3D shape, (iii) the hit and negative compounds had similar distributions of sp^3^-hybridized carbon atoms, (iv) 3D descriptors are sensitive to conformation, and the assay used in this study could not determine the bioactive conformation of molecules, and (v) the use of conformer ensembles is not rigorous enough in determining the true association between the observed signal and the underlying biology.^26,27^

Selected models were used to prospectively predict oxidative stress for a collection of new compounds to further assess the predictive performance of the models generated in this exercise. A significant effort was made to prospectively select representative compounds that would reflect a “real-world” test set. For this, a set of 160 compounds was designed and obtained from the Enamine HTS collection of 1,815,615 compounds with the aim to verify model predictions through experimental validation.

Predictions for 20,000 compounds were computed, and 160 compounds were selected for experimental testing based on the maximum combined prediction confidence and based on similarity (high and low) to our modeling dataset and chemical space clustering. This scheme was chosen to obtain insights both into model performance and the importance of compound similarity to our training series and chemical space. This work not only resulted in a valuable set of oxidative stress predictive models but also served as an example of a comprehensive cheminformatics analysis and modeling exercise from experimental design and testing to prospective validation.

## Methods

### Cell Culture and Reagents

Previously, a bacterial artificial chromosome (BAC) containing the mouse sulfiredoxin (Srxn1) gene under the control of the endogenous promotor was cloned with green fluorescent protein (GFP) to create an Srxn1-GFP fusion protein.^17^ The Srxn1-GFP BAC was transfected and stably integrated into an ATCC (clone HB8065) human hepatoma HepG2 cell line. The HepG2 Srxn1-GFP line responds to oxidative stress-inducing compounds, thus functioning as an oxidative stress reporter cell line. In this work, HepG2 Srxn1-GFP cells were cultured in Dulbecco’s modified Eagle’s medium (DMEM), high glucose supplemented with 10% (v/v) fetal bovine serum (FBS), 25 U/mL penicillin, and 25 μg/mL streptomycin. The cells were used between passages 5 and 20. For live cell imaging, the cells were seeded in Greiner black μ-clear 96 wells plates at 20,000 cells per well. Compounds of the Spectrum Library acquired at a concentration of 10 mM were diluted in dimethyl sulfoxide (DMSO) at a concentration of 10 μM, allowing to keep the DMSO concentration to a maximum of 0.1%.

### Exposure and Microscopy

The HepG2 Srxn1-GFP BAC reporter cell line was exposed to 30 nM CDDO-Me as the NRF2 activating entity, leaving a dynamic range for up- and downregulation by the Spectrum Library compounds with respect to the CDDO-Me baseline activation. 10 μM Spectrum Library compounds were added in three replicate plates, which were incubated for 24 h. Such concentration was chosen as it corresponds to that used for 94% of the compounds (150/158) tested in PHH in TG-GATEs—a library designed to include compounds involved in liver toxicity and covering several stress mechanisms, including oxidative stress.^28^ Additionally, a 24 h exposure window was chosen as it corresponds to the time at which maximum response is observed in this reporter.^29,30^ After 24 h, the plates were fixed with formaldehyde and stained with the nuclear dye Hoechst 33258. GFP intensity levels were imaged with a Nikon TiE2000 confocal laser scanning microscope (lasers: 408 and 488 and 20× magnification).

### Quantitative Image Analysis

Individual cells were identified using the nuclear Hoechst staining, and attached cytoplasmic GFP intensity levels were analyzed with Cell Profiler version 2.1.1^31^ and were subsequently processed as previously reported.^17^ Images in which either less than 100 cells were present or with a GFP intensity signal greater than three times the median absolute deviation of their respective plate were not considered. GFP intensity values were converted to a modified *Z*-score such that modified *Z*-score = ((*x* – *X̃*))/(*k* × MAD), with *X̃* the median, MAD the median absolute deviation, and *k* = 1.4826. Subsequently, the median of the modified *Z*-score of experimental repeats was used as the dependent variable for modeling. A median-modified *Z*-score value of over 1.96 was defined as active in this dataset.

### Modeling

#### Spectrum Library: Training and Test Sets

The Spectrum Collection compound library (Microsource Discovery Systems) consists of 2230 compounds and includes compounds that reached clinical trials in the U.S. (U.S. Drug Collection), drugs marketed in Europe and/or Asia that were not introduced to the U.S. (International Drug Collection), and natural products and derivatives of plant, animal, and microbial sources (Natural Product Collection) and compounds that despite having shown biological activity in peer-reviewed publications were never developed as treatments for human diseases (Discover Collection). Ionized chemical structures were neutralized, and counterions were removed. Inorganics, organometallics, and mixtures were discarded. Data from the curation procedure was gathered in a dataset of 2191 compounds, including 316 positive and 1875 negative compounds, and were used for modeling median-modified *Z*-scores, hereafter referred to as activities. A training set (1520 compounds, 218 actives) and a test set (671 compounds, 98 actives) were derived. The splitting strategy involved the clustering of structures using the affinity propagation method (as implemented in Pipeline Pilot) using functional circular fingerprints with radius 2 (FCFP_4), with proportionate stratified random sampling (70 and 30% for training and test sets, respectively). Subsequently, the training set was distributed to all partners with the bioactivity measures, while the test set was distributed blindly.

The splitting method proved to be robust, and chemical structure distribution was consistent with the observed bioactivity. To validate this, the bioactivity of the measured nearest neighbors from the test set was compared using functional circular fingerprints with radius 3 and 2048 bits (FCFP_6) to that of compounds in the training set (Table 1). In 80.65% of the cases, the biological activity was the same (nearest neighbors of active compounds were active and vice versa), only 10.99% of active compounds of the training set had inactive nearest neighbors in the test set, and only 8.36% of inactive compounds of the training set had active nearest neighbors in the test set.

**Table 1:** Confusion Matrix of Experimental Activities of the Test Set Compounds Compared to Their Nearest Neighbors Present in the Training Set

		activity of nearest neighbor in the training set	
		active	inactive
activity in the test set	active	51	167
	inactive	127	1175

#### Machine Learning Models

Each partner organization developed its own set of statistical machine learning structure–activity relationship (SAR) models. Though varying methodologies were employed to develop such models, all of them were fitted on the same training subset of the Spectrum Library (Figure S1).

Five classifiers were built using the eTOXlab^32^ under a conformal prediction (CP) framework^33^ to determine each model’s applicability domain. A random forest^34^ (RF) and a support vector machine (SVM) model were built based on RDKit Morgan fingerprints^35,36^ (UPF 1 and 2 in Table 3, respectively). Additionally, a partial least-squares regression (PLSR) model was developed using Adriana-Code descriptors (UPF 3) and two RF models using Padel^37^ and VolSurf^38^ molecular descriptors, respectively (UPF 4 and 5).

Four RF models were developed under the Mondrian conformal prediction (MCP) framework,^33^ using the nonconformist Python package. Well-calibrated *p*-values were obtained for the assignment to the active or inactive classes and to determine the applicability domains of the two models. The models were evaluated at significance levels of 0.25 and 0.30. Two models were developed using signature fingerprints^39^ (Swetox 1 and 2, respectively), and two others were based on RDKit physicochemical molecular descriptors (Swetox 3 and 4, respectively).

A balanced random forest (BRF)^40^ model (MN 8), as implemented in the KNIME Analytics Platform,^41^ was derived from Dragon (v. 7.0.8, Kode SRL, 2017) molecular descriptors. This type of model alters the class distribution so that classes are represented equally in each tree. Descriptors were pruned by constant and semi-constant values and, should pairs of descriptors have an absolute correlation higher than 90%, only one descriptor was retained.

Three classifiers were derived from Pipeline Pilot FCFP_6.^35,42^ One was a naive Bayes (NB) model, and the two others consisted of *k*-nearest neighbors (*k*NN) classifiers developed using either one single or three nearest neighbors (UL 5, 12, and 13, respectively).

Four classifiers were derived from the combination of Pipeline Pilot FCFP_6 fingerprints and physicochemical descriptors. These consisted of a PLSR, an RF, an SVM, and a logistic regression (LR) model (UL 7, 8, 9, and 4, respectively).

Two deep neural networks (DNN) were trained (UL 3 and 1, respectively) using either only RDKit Morgan fingerprints with radius 3 or combined with RDKit physicochemical descriptors (PhysChem).

Three classifiers were derived from bioactivity spectra (BS) derived from previously published NB and DNN classifiers.^43^ Although the predicted bioactivities of one target might not scale as that of another target, similar patterns of activities are associated with similar endpoints. Such BS have demonstrated increased performance in predicting complex endpoints, along with cellular responses and clinical outcomes.^44^ The first model was an NB model derived solely from BS (UL 6), while the two others consisted of RF models trained on BS combined with Pipeline Pilot FCFP_6 fingerprints and physicochemical descriptors (UL 10 and 11, respectively).

Two models were developed with SARpy.^45^ SARpy extracts rules after having fragmented input molecular structures and searches for relationships between the generated fragments and the observed activity. SARpy was used to search for fragments specific to the active and inactive classes (MN 5) or specific to the inactives only (MN 6).

One model was trained on randomized input descriptors and used as a baseline random estimator (UL 14).

Four classifiers were obtained by subdividing the original training set into a training subset (1215 compounds, 80% of the initial dataset) and a validation subset (305 compounds, 20% of the initial dataset). The splitting was performed by *k*-means clustering considering the mean Tanimoto similarity of each compound with respect to other compounds in the dataset and to their activity in order to guarantee a uniform structural and activity distribution between the two datasets. Subsequently, to adjust for the unbalanced distribution of classes, the training subset was under-sampled by deleting the most represented class (i.e., inactive compounds) until both classes were equal in number. The same *k*-means clustering method used for splitting the original training set was used for this under-sampling in order to keep a fraction of chemicals representative of the full set of negative compounds. In the end, a final balanced training subset of 348 compounds was obtained. Classification models were derived from this final balanced training subset, the first of which was derived from CORrealtions And Logic (CORAL) software^46^ (MN 2). CORAL derives optimal descriptors from SMILES, i.e., attributes that check the presence of particular characters or combinations of them. Other models consisted of an RF model and a decision tree (DT),^47^ derived from Dragon descriptors (MN 7 and 3, respectively). The last model was a gradient-boosted tree (GBT) based on ensemble modeling and a boosting strategy^48^ (MN 4). These RF, DT, and GBT models were developed using the KNIME Analytics Platform.

Finally, four ensemble models were trained. The first consisted of a majority vote strategy ensemble model (MN 1) and was derived from models MN 2, 3, 4, 5, 6, and 7. In particular, a compound was classified as positive or negative if at least five out of six models had concordant predicted labels; otherwise, the compound was flagged as suspicious. The second ensemble model (UL 2) was derived from all of the 30 aforementioned models, including the MN 1 ensemble model, and consisted of a majority vote strategy ensemble model based on predicted labels. The third and fourth ensemble models relied on the average and median of the predicted probabilities from all of the 31 aforementioned models, including the MN 1 and UL 2 models (Ensemble mean and Ensemble median, respectively).

### Selection of Compounds for Prospective Validation

The Enamine HTS collection (downloaded in August 2017), containing 1,815,615 diverse screening compounds, was selected for virtual screening. The collection encompasses versatile chemotypes developed within a couple of decades of chemical research at Enamine and its partner academic organizations. These compounds frequently have singular structures and unique properties, making them an ideal diverse screening set. The activity of the compounds was predicted by SARs models selected based on their performance on the holdout test set. Since the in vitro validation of predicted activities of 1,815,615 compounds was not feasible, a subselection of the Enamine HTS collection based on subregions of a reference chemical space was devised.

#### Reference Chemical Space

A reference chemical space was created with the aim to select representative compounds from the Enamine HTS Collection to use for prospective validation of the developed models on chemical structures closely related to those of industrial and real-life interest. More specifically, the reference chemical space was defined by gathering chemicals from three different datasets: COSMOS,^49^ as it is representative of substances of toxicological concern, DrugBank,^50^ representative of approved drugs and nutraceuticals, and the annex VI of the classification, labeling, and packaging (CLP) regulation of the European Chemicals Agency (ECHA) on chemical substances representative of industrial chemicals (Table 5). Compounds from the three datasets were standardized, and functional Morgan circular fingerprints with radius 2 folded to 1024 bits were computed using RDKit.^36,51^ Principal components (PC) analysis with two components was applied to the descriptor matrix centered and scaled to unit variance beforehand. The PCA scores were then clustered using *k*-means with 6 clusters, 100 seeds, and the *k*-means++ algorithm for centroid initialization.^52^ The obtained two-dimensional (2D) chemical space defined by the PC alongside the obtained clusters (Figure 2) was used to guide the selection of compounds whose activity was to be validated in vitro.

#### Classification of Enamine Compounds

The compounds of the Enamine dataset were characterized by their similarity with respect to the training set and location in the reference chemical space using functional Morgan circular fingerprints with radius 2 folded to 1024 bits. First, Enamine compounds were classified based on Tanimoto similarity (Tc) as similar (Tc ≥ 0.7) and dissimilar (Tc ≤ 0.3) from the modeling dataset. Subsequently, each similarity group was projected and classified into the corresponding clusters of the reference set (Table 2). The 482,306 compounds classified as dissimilar were randomly subsampled to 20,000.

**Table 2:** Cluster Population for the Considered Selections^a^2

	cluster					
	0	1	2	3	4	5
	purple	red	green	cyan	indigo	yellow
similar compounds	172	2290	23	372	943	1
dissimilar compounds	4460	2574	200	805	11,961	0

a For dissimilar selection (Tanimoto similarity ≤0.3), the number of compounds was randomly subsampled to 20,000. The cluster numbers and colors correspond to that in Figure 2.

#### Compound Selection

The bioactivities of the 23,801 Enamine compounds previously selected based on subregions of the reference chemical space were predicted using the models described above. To narrow down the selection of compounds, the concordance in terms of predicted labels among models was evaluated alongside inclusion in their applicability domains. Four categories of compounds were scrutinized based on their similarity group and predicted activity. Similarities were evaluated with Tanimoto coefficients (Tc) derived from functional Morgan circular fingerprints with radius 2 folded to 1024 bits. Compounds predicted as active and similar to the modeling set were included if at least 70% of models were concordant and if they were included in the applicability domains of at least 70% of models. For compounds predicted as active and dissimilar to the modeling set, this threshold was increased to 80%. This threshold was further tuned for compounds predicted as inactive and was set to 90% for those similar to the training set and to 100% for those dissimilar to the training set. The higher threshold picked for consensual prediction of inactive compounds results from the higher occurrence of inactives in the training set. Subsequently, compounds were assigned to their similarity classes (similar or dissimilar), predicted activity classes (active or inactive), and PCA clusters of the chemical space (clusters 0–5), resulting in 24 different compound groups. Compound groups populated with less than 25 members were considered for purchase and in vitro validation. Complementarily, groups populated with more than 25 compounds were clustered into 5 subclusters using k-means. From each subcluster, 5 compounds were taken summing up to at most 25 compounds to be tested per cluster, similarity, and predicted activity groups.

## Results

Experimental data was distributed among four different modeling groups, partners of the EU-ToxRisk Consortium. Each partner built different prediction models using their own methodologies. The models’ predictive performance was evaluated using a common test set blinded before initiating the modeling work. The predictive quality and orthogonality of predictions were the parameters chosen to select models, while models that were either not predictive or whose predictions were very similar to other models were discarded. Only two-dimensional (2D) molecular descriptors were considered, as there were no significant differences between positive and negative hit compounds (Figures S2 and S3).

### Modeling Results

Model performances on the holdout test set are summarized in Table 3 (complete overview in Table S1). Generally, models show a tendency to produce false negatives (FN) and, therefore, to have low sensitivity, as expected, given the dataset imbalance. Taking Matthews correlation coefficient^53^ (MCC) as a performance index, models demonstrated to be predictive with an average MCC of 0.23. The additional random predictor included as a baseline with an MCC of 0.05 shows the enriched predictive power of the models. Subsampling strategies were used in some of the models achieving a more balanced difference between sensitivity and specificity at the expense of a reduced applicability domain coverage. Interestingly, the consensus model MN 1 achieved a remarkable performance with an MCC of 0.44, yet with a drop in the coverage of its applicability domain (52%).

**Table 3:** Model Performance on the Holdout Test Set Ranked by MCC^a^

model name	algorithm	descriptors	SN	SP	ACC	BACC	AD	MCC
perfect	-	-	1.00	1.00	1.00	1.00	1.00	1.00
MN 1	consensus	various	0.65	0.87	0.84	**0.76**	0.52	**0.44**
Swetox 4	CP/RF	RDKit	0.71	0.72	0.72	0.72	0.79	0.31
UL 10	RF	PP FP, BS, PhysChem	0.51	0.84	0.79	0.68	1.00	0.30
ensemble	mean	-	0.47	0.86	0.80	0.66	1.00	0.29
ensemble	median	-	0.45	0.87	0.81	0.66	1.00	0.29
Swetox 2	CP/RF	signatures	0.65	0.74	0.72	0.70	0.76	0.29
UL 8	RF	PP FP, PhysChem	0.51	0.83	0.78	0.67	1.00	0.28
UPF 1	CP/RF	RDKit FP	0.63	0.72	0.71	0.68	0.68	0.26
Swetox 3	CP/RF	RDKit PhysChem	0.67	0.70	0.70	0.69	0.91	0.26
UL 5	NB	PP FP	0.68	0.68	0.68	0.68	1.00	0.26
UPF 2	CP/SVM	RDkit FP	0.71	0.61	0.66	0.66	0.67	0.26
MN 8	BRF	Dragon	0.44	0.85	0.79	0.65	1.00	0.25
UL 6	NB	BS	0.42	0.86	0.79	0.64	1.00	0.25
UPF 3	CP/PLSR	Adriana	0.70	0.66	0.66	0.68	0.71	0.25
UL 3	DNN	RDKit FP	0.27	0.93	0.84	0.60	1.00	0.24
UPF 4	CP/RF	PaDEL	0.67	0.67	0.67	0.67	0.75	0.24
UL 11	RF	PP FP, BS, PhysChem	**0.71**	0.62	0.63	0.67	1.00	0.24
MN 4	GBT	Dragon	0.60	0.71	0.69	0.66	1.00	0.23
UL 13	kNN (3NNs)	PP FP	0.19	0.96	0.85	0.58	1.00	0.22
MN 7	RF	Dragon	0.54	0.74	0.71	0.64	1.00	0.22
UL 12	kNN (1NN)	PP FP	0.31	0.89	0.81	0.60	1.00	0.21
UPF 5	CP/RF	VolSurf	0.57	0.72	0.70	0.65	0.74	0.21
Swetox 1	CP/RF	signatures	0.58	0.70	0.68	0.64	0.89	0.21
MN 5	SARpy	SAs (actives, inactive)	0.47	0.77	0.73	0.62	0.99	0.20
MN 6	SARpy	SAs (inactives)	0.61	0.64	0.64	0.63	1.00	0.18
UL 2	majority vote	various	0.34	0.85	0.77	0.60	1.00	0.17
UL 1	DNN	RDKit FP, PhysChem	0.61	0.62	0.62	0.62	1.00	0.17
MN 2	CORAL	SMILES-based	0.71	0.52	0.55	0.62	0.92	0.16
UL 7	PLSR	PP FP, PhysChem	0.19	0.92	0.82	0.56	1.00	0.14
UL 9	SVM	PP FP, PhysChem	0.03	**1.00**	**0.86**	0.52	1.00	0.13
MN 3	DT	Dragon	0.57	0.61	0.60	0.59	1.00	0.13
UL 4	LR	PP, FP, PhysChem	0.36	0.77	0.71	0.57	1.00	0.11
UL 14	random	randomized	0.55	0.52	0.52	0.54	1.00	0.05

a The performance of a perfect model is given for comparison. The best values for sensitivity (SN), specificity (SP), accuracy (ACC), balanced accuracy (BACC), and Matthews correlation coefficient (MCC) are highlighted in bold. AD stands for the coverage of the applicability domain, PP FP for Pipeline Pilot FCFP_6 fingerprint, and NN for nearest neighbor.

### Error Analysis

**Figure 1:**
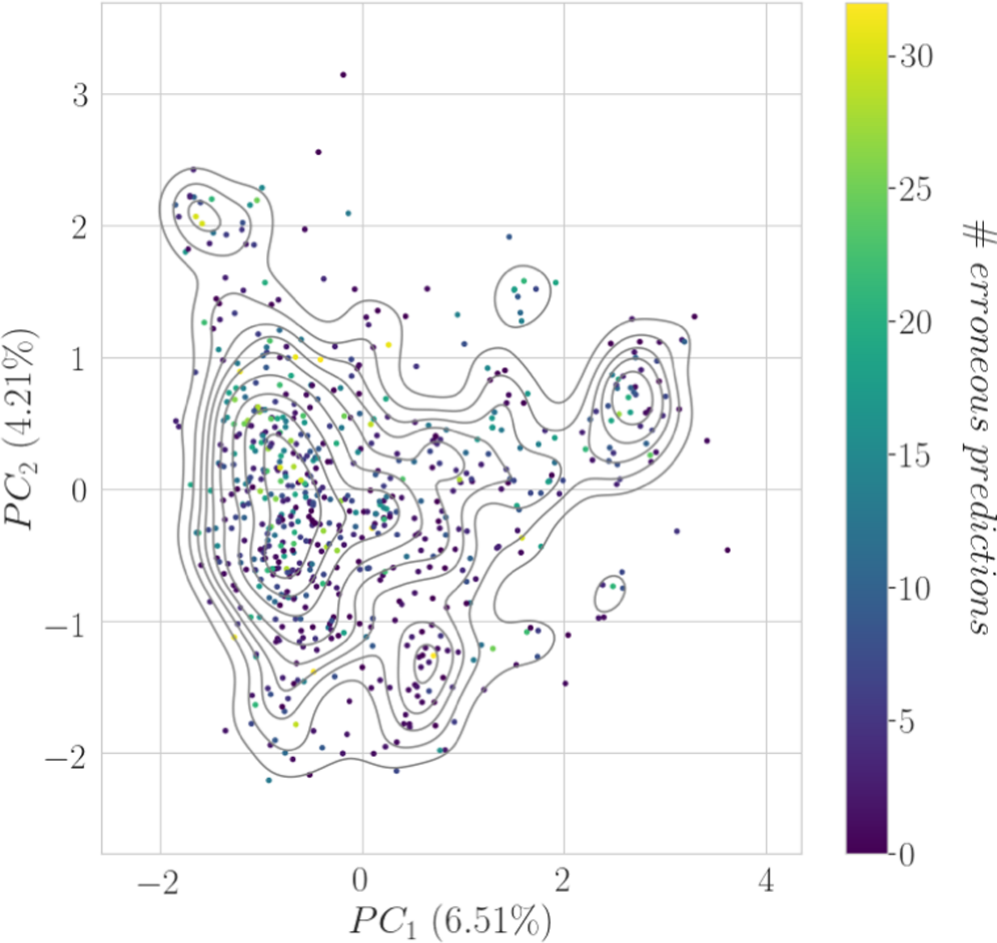
Distribution of the number of erroneous predictions made by models reported in Table 3 in the PCA space of the Spectrum holdout test set.

Identifying the reasons why some of the predictions with a higher agreement are erroneous is essential to understand the limitations of the models. The sum of erroneous model predictions per compound in the holdout test set was investigated (Figure 1). Compounds associated with more mispredictions were distributed proportionally according to the number of compounds in their vicinity in the chemical space (based on Tanimoto similarity of functional circular fingerprints with radius 2; FCFP_4). This phenomenon rejects the possibility of specific chemical properties being the cause of such errors and can be attributed both to the diverse nature of the Spectrum dataset and to the variety of mechanisms involved in oxidative stress, which can be triggered by a wide diversity of chemicals themselves.

Some of the compounds presented a high consensus in incorrect predictions and were further analyzed. For each of them, the five most similar compounds, in terms of the Tanimoto coefficient based on 2048 bits FCFP_4, from the training dataset, were extracted (Table 4; detailed information in Table S2). These compounds were considered as being the most associated with incorrect predictions, and their analysis led to the identification of the following sources of errors.

**Table 4:**
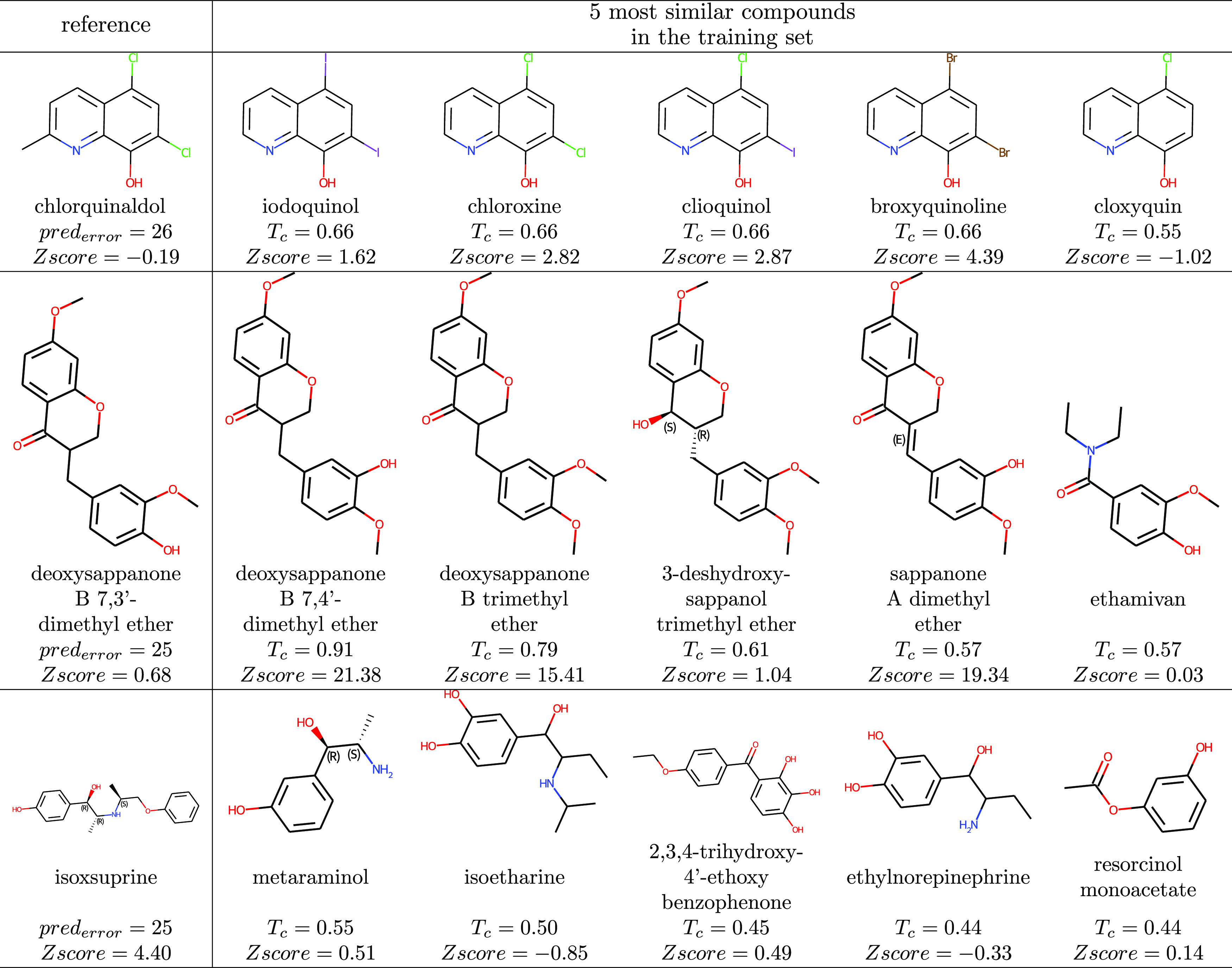
Consensually Mispredicted Compounds^a^

a Compounds of the blinded holdout test set associated with highly consensual mispredictions (pred_error_) are denoted as references. Their closest homologues in the training set are depicted alongside their Tanimoto similarity (Tc) and median-modified *Z*-score (*Z*score).

#### Activities Close to Neutral

The binary labels the models were fitted to were derived from *Z*-scored GFP intensity values. Though the threshold used was quite conservative when it comes to activity, the strength of the effect of compounds on the oxidative pathway may differ substantially. Thus, *Z*-scores of similar compounds in a chemical series can oscillate around the activity threshold. For example, chlorquinaldol was predicted as active by 26 models, while the experimental result is inactive (*Z*-score of −0.19). The five most similar compounds (iodoquinol, chloroxine, clioquinol, broxyquinoline, and cloxyquin) have a very similar scaffold with distinct substituents. The experimental activities for these compounds show *Z*-score values close to the activity threshold, except for broxyquinoline’s, which is 4.40.

#### No Representative Compound in the Training Set

Due to the chemical diversity of the Spectrum Library, compounds in the test set might not be represented in the training set. If so, important features of a particular compound series for their activity might not be considered by the algorithms, resulting in incorrect labels. For example, isoxsuprine’s most similar compound, metaraminol, is dissimilar with a Tanimoto similarity of 0.55.

#### Mechanism Not Captured

Another source of errors is the difficulty for the algorithms to identify the underlying mechanisms from the given descriptors. For example, deoxysappanone B 7,3′-dimethyl ether and deoxysappanone B 7,4′-dimethyl ether, though having 0.91 Tanimoto similarity, show an activity cliff with *Z*-scores of 0.68 and 21.38, respectively, hence being respectively inactive and active. The subtle structural difference corresponding to alternated hydroxy and methoxy groups makes most models predict it as active.

### Model Selection

Of the 33 models developed, only the best-performing models in terms of MCC and balanced accuracy were selected. Additionally, any model with either sensitivity or specificity lower than 0.50 was disregarded. For the models developed by IRFMN, this resulted in models MN 1 and MN 4 being selected. Models 1 and 3 developed by Swetox were selected for the larger coverage of their applicability domains (0.89 and 0.91, respectively) compared with models Swetox 2 and Swetox 4 (0.76 and 0.79, respectively), though compromising for lower MCC (0.64, 0.69, 0.76 and 0.79, respectively). Of the models developed by UL, models 1, 5, 8, and 11 were selected, preferring model UL 11 over UL 10 for its more balanced sensitivity and specificity (0.71 and 0.62 against 0.51 and 0.79, respectively). Finally, models 1, 3, and 4 developed by UPF and the mean ensemble model were selected. A consensus majority vote model was devised based on the 11 selected models.

### Prospective Validation

Estimating the true quality of a model is a challenging task. Results obtained using a test set too similar to the training series can give an overly optimistic estimation of model performance. Conversely, if too different from the training series, that is, containing compounds out of the chemical space of interest, a test set can produce pessimistic estimations. In this work, a realistic validation exercise was devised by carefully extracting a validation set from the “chemical space of interest.” The similarity to the training series and the quality of the predictions were also included as selection criteria. Additionally, the validation of models’ predictions was conducted prospectively to avoid any involuntary bias.

The reference chemical space of interest was obtained by integrating source datasets of different natures: DrugBank accounted for the druggable chemical space and COSMOS and ECHA’s annex IV for the toxicity-associated chemical space (Table 5). The chemical space was then clustered into six regions.

**Table 5:** Datasets Used for Building a Representative Chemical Space

source	no. unique molecules	origin
COSMOS	42,935	substances present in cosmetic products
DrugBank	7225	approved small molecule drugs, nutraceuticals, and experimental drugs
ECHA CLP annexe VI	2384	substances classified as hazardous by ECHA

**Figure 2:**
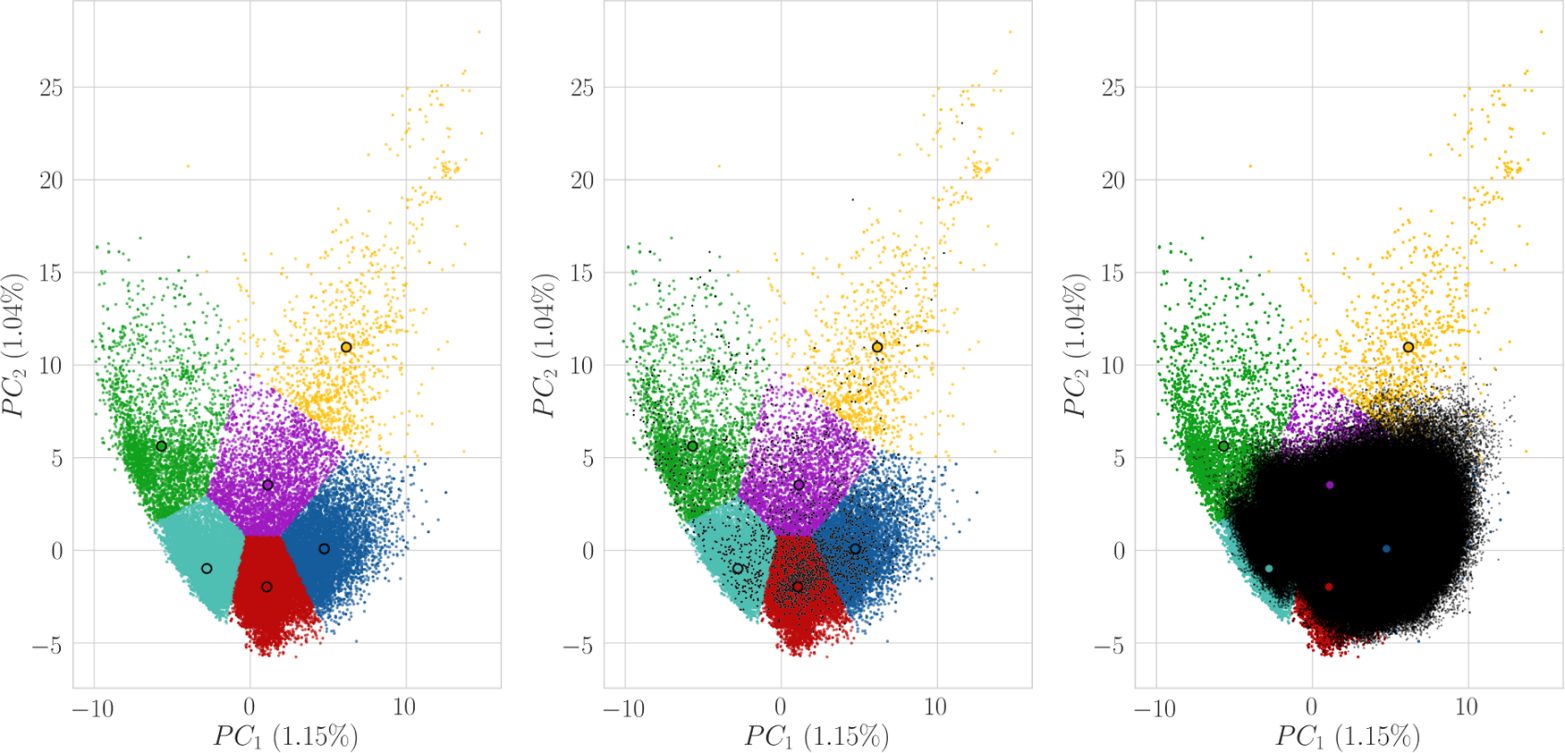
Reference clustered chemical space (A) with projections of the Spectrum Library (B) and Enamine HTS collection (C). Clusters and centers are represented in color, and projected datasets are represented in black. Clusters 0–5 are represented in purple, red, green, cyan, indigo, and yellow, respectively.

**Figure 3:**
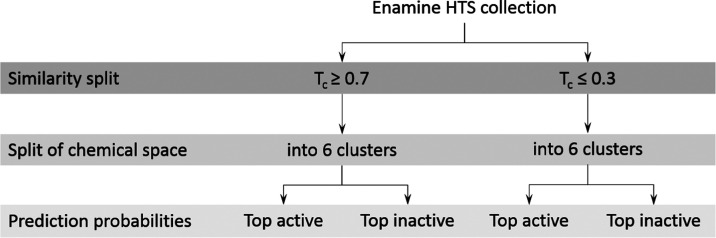
Schematic representation of the prospective candidate selection. First, Enamine compounds were classified by Tanimoto similarity (Tc) with respect to the Spectrum training set into two groups; similarity equal to or greater than 0.7 as the similar group, and similarity equal to or lower than 0.3. Compounds were further projected in the reference chemical space and classified upon the cluster they fell into. Finally, compounds with the top probabilities of being active and inactive in each subgroup were considered for experimental validation.

The Enamine HTS database was selected for the prospective selection due to its chemical diversity. The database, once standardized, contained 1,815,615 compounds. These compounds were projected into the reference chemical space and then classified according to the six clusters they fell into (Figure 2) and according to their similarity to the training series (Figure 3). Such a selection ensured the obtention of valuable information on the influence of both similarity and chemical space localization on the models’ performance.

The 11 selected models, along with the consensus model, were then used to predict oxidative stress for the highest probabilities of being active and inactive, resulting in a list of 23,801 molecules. From them, 20,194 remained after some were rejected through errors occurring either during standardization or during the computation of molecular descriptors. Subsequently, at most 25 compounds per cluster, similarity, and predicted activity groups were selected. Table 6 summarizes the distribution of compounds selected within clusters of the chemical space. The final list of compounds selected for the prospective validation is available in the Supporting Information.

**Table 6:** Distribution of Compounds within Similarity Clusters Selected for Prospective Validation

	cluster						
	0	1	2	3	4	5	total
similars predicted as active	-	25	-	-	24	-	49
similars predicted as inactive	2	15	5	12	13	-	47
dissimilars predicted as active	7	9	-	2	14	-	32
dissimilars predicted as inactive	12	3	1	5	10	1	32

### Prospective Results

The 160 selected compounds were then validated in vitro, and the measured activation of the oxidative stress pathway was compared to the models’ predictions (Table 7). A small decrease in average performance, with an MCC of 0.20 and accuracy of 0.61, was observed when compared to that of the holdout test set (0.23 and 0.72, respectively). This originated from the filtering out of models with unbalanced sensitivity and specificity, introducing a false positive bias in the models, hence translating in lower general performance when compared to the performance of the prospective validation set. Nevertheless, the models were able to identify 15 oxidative stress-inducing compounds out of the 19 measured. This result highlights the capacity of the models to identify positive compounds from a library of samples despite a small positive-to-negative ratio (herein 1:7.42) due to the complexity of the endpoint scrutinized, whose inner mechanisms are not fully captured. Yet, models with very high sensitivity are preferred, regardless of specificity, as they have a low risk of missing potentially hazardous compounds. Interestingly, the consensus model was the second-best in terms of sensitivity (0.79) but penultimate in terms of specificity (0.53), resulting in a low MCC of 0.21 and disappointing performance.

**Table 7:** Performance of Models on the Prospective Validation Set^a^

model name	SN	SP	ACC	BACC	AD	MCC
UPF 1	0.63	0.78	0.65	**0.70**	1.00	**0.27**
MN 1	0.74	0.58	0.72	0.66	0.71	0.22
UL 5	0.55	0.79	0.58	0.67	1.00	0.22
UPF 4	0.56	**0.80**	0.58	0.68	1.00	0.22
UL 11	0.54	0.79	0.57	0.66	1.00	0.21
consensus	0.79	0.53	0.56	0.66	0.97	0.21
MN 4	0.52	0.79	0.56	0.66	1.00	0.20
Swetox 3	0.51	0.79	0.54	0.65	0.99	0.20
Swetox 1	0.57	0.72	0.59	0.65	0.94	0.19
UPF 3	0.55	0.74	0.57	0.64	1.00	0.18
UL 8	0.53	0.74	0.56	0.63	1.00	0.17
UL 1	**0.86**	0.32	**0.79**	0.59	1.00	0.15

a Models are sorted by MCC. The best values for sensitivity (SN), specificity (SP), accuracy (ACC), balanced accuracy (BACC), and Matthews correlation coefficient (MCC) are highlighted in bold. AD stands for coverage of the applicability domain.

#### Performance by Cluster

Interestingly, the performance of models was not evenly distributed along the chemical space (Table 8; complete overview in Table S3). This was reflected by clusters 2 and 5, consisting of only negative compounds, where specificities of 1.00 were observed for all models but one (MN 4) in the case of cluster 2. Though the prospective validation set contained mostly inactive compounds, with only 19 actives out of the 160 tested, the best performances in terms of MCC were obtained within clusters 0 and 3 (0.32 and 0.27, respectively). Nevertheless, for the latter, models had both unequal predictive power as demonstrated by the increased standard deviation of the MCC and a decreased average sensitivity (0.1 and 0.30, respectively) when compared to clusters 0, 1, and 4. Clusters 1 and 4 had higher average balanced accuracies than cluster 3 (0.64, 0.68, and 0.61, respectively) due to their higher average sensitivities (0.82 and 0.85 against 0.30, respectively) but had the lowest average MCC (0.21 and 0.20, respectively), explained by the lower average specificities (0.46 and 0.50, respectively).

**Table 8:** Average Performance of the Models on the Prospective Validation Set per Cluster^a^

cluster	no. actives/no. inactives	SN	SP	ACC	BACC	MCC
0	2.82 ± 0.40:17.82 ± 0.60	0.61 ± 0.11	0.77 ± 0.09	0.75 ± 0.07	0.69 ± 0.04	0.32 ± 0.11
1	7.73 ± 0.90:42.73 ± 2.45	0.82 ± 0.13	0.46 ± 0.11	0.51 ± 0.08	0.64 ± 0.04	0.21 ± 0.05
2	0.00 ± 0.00:6.00 ± 0.00	0.00 ± 0.00	0.98 ± 0.05	0.98 ± 0.05	NA	0.00 ± 0.00
3	3.00 ± 0.00:15.73 ± 0.65	0.30 ± 0.18	0.93 ± 0.05	0.82 ± 0.04	0.61 ± 0.08	0.27 ± 0.21
4	4.91 ± 0.30:54.09 ± 3.27	0.85 ± 0.28	0.50 ± 0.16	0.53 ± 0.13	0.68 ± 0.08	0.20 ± 0.08
5	0.00 ± 0.00:1.00 ± 0.00	0.00 ± 0.00	1.00 ± 0.00	1.00 ± 0.00	NA	0.00 ± 0.00

a Models are sorted by MCC per cluster. Values are reported as mean ± standard deviation across models for the compounds of the denoted cluster. No. actives, No. inactives, SN, SP, ACC, BACC, and MCC stand for the number of active and inactive compounds falling within each model’s applicability domain, sensitivity, specificity, accuracy, balanced accuracy, and Matthews correlation coefficient, respectively. NA stands for values that could not be determined due to the models’ lack of sensitivity.

#### Log *P* and Performance

The *n*-octanol/water partition coefficient (Log *P*), together with the dose, had previously been reported to distinguish compounds associated with DILI from others.^54,55^ A balanced Log *P* favors drug solubility in the serum and facilitates cell uptake through membrane diffusion. To assess the bias of the models toward high Log *P* values, the performance of the models was also assessed by separating the compounds in four intervals of Crippen’s atom-based approximation of Log *P*:^56^ Log *P* ≤ 0, 0 < Log *P* ≤ 2.5, 2.5 < Log *P* ≤ 5, and Log *P* ≥ 5 (Table 9; complete overview in Table S4). Log *P* was computed for the prospective dataset using RDKit. Compounds with a Log *P* below 2.5 were mostly negative (60 out of 63), with three exceptions, probably due to facilitated transport processes. This result highlights the predictive power of the sole Log *P* descriptor for negative compounds. On the other hand, most of the compounds with a Log *P* in the range of 2.5–5 can reach the cell cytoplasm due to favorable physicochemical properties, though other phenomena such as metabolism might be at play. Most positive oxidative stress inducers (15 out of 19) were found in this group. The models were able to correctly identify 12 of the 15 active and 22 of the 68 inactive compounds. These results highlight the limitation of the models to capture the underlying mechanisms leading to oxidative stress.

**Table 9:** Average Performance of the Models on the Prospective Validation Set per Log *P* Interval^a^

interval	no. actives/no. inactives	SN	SP	ACC	BACC	MCC
Log *P* ≤ 0	2.00 ± 0.00:10.82 ± 0.60	0.05 ± 0.15	0.98 ± 0.05	0.84 ± 0.02	0.51 ± 0.05	0.02 ± 0.08
0 < Log *P* ≤ 2.5	1.00 ± 0.00:48.73 ± 0.47	0.00 ± 0.00	0.99 ± 0.02	0.97 ± 0.02	0.49 ± 0.01	–0.01 ± 0.01
2.5 < Log *P* ≤ 5	14.55 ± 1.21:65.45 ± 4.46	0.84 ± 0.17	0.33 ± 0.18	0.42 ± 0.12	0.58 ± 0.03	0.15 ± 0.05
5 < Log *P*	0.91 ± 0.30:12.36 ± 1.80	0.82 ± 0.40	0.12 ± 0.22	0.18 ± 0.20	0.52 ± 0.09	0.03 ± 0.10

a Models are sorted by MCC per Log *P* interval. Values are reported as mean ± standard deviation across models for the compounds of the denoted Log *P* interval. No. actives, No. inactives, SN, SP, ACC, BACC, and MCC stand for the number of active and inactive compounds falling within each model’s applicability domain, sensitivity, specificity, accuracy, balanced accuracy, and Matthews correlation coefficient, respectively.

## Discussion

From the initial stages of building a model to its deployment for real-life applications, many factors have to be studied to ensure the model’s validity. This analysis is of the utmost importance when the objective function is rather abstract, for example, when all possible biological adverse outcomes the studied chemicals could trigger are not fully known or when the underlying biological mechanisms are only partially understood. Oxidative stress is a good example as it is a complex phenomenon that results from the interplay of multifaceted pathways whose mechanisms are yet to be completely unraveled.^57^ Thus, the nonlinear relationships between molecular features and the biological response a model would need to identify are amplified due to these intricacies.

Another critical factor when developing a machine learning model resides in the data used to fit the algorithm. The Spectrum Library was selected herein due to its high molecular diversity, hence covering a large chemical space. However, this large diversity comes with its drawbacks. For example, the compounds might not be evenly distributed among different biological mechanisms, hence biasing the algorithms toward the most representative mechanisms, especially if the molecular descriptions are not complex enough.^58^ In this case, the underlying machine learning algorithms will fail to find complex patterns differentiating between mechanisms but will rely on simpler associations, resulting in lower performance on the less represented mechanisms and will have lower generalization capacity.

In this work, we have addressed the modeling of oxidative stress from the early stage of model creation up to its application in a prospective experimental validation of the selected compounds. Modeling was approached by letting the different academic partners use their own modeling methodologies—descriptors and algorithms—on the same training set. The development of classifiers was favored over regression models as the latter would not have worked as well, considering the diversity of the chemicals in the training set and the inherent difficulty of developing predictive regression models. The variability in modeling and subsampling techniques favored also the variability of predictions and applicability domains. In turn, this variability of predictions translated into a panel of models with heterogeneous performance. In general, models showed higher specificity than sensitivity, especially those models in which balancing approaches were not adopted. From the disparate trends in model performances, one could wonder which models to prioritize for the prediction of oxidative stress in a real-life scenario. Models with high specificity and low sensitivity would certainly predict most of the compounds correctly since most drug- and lead-like compounds are not expected to show oxidative stress activity. However, such models would be noninformative due to their inability to identify active compounds. On the other hand, models with more equilibrated sensitivities and specificities would have a higher false positive rate and thus be biased toward active predictions. Additionally, most models with balanced sensitivities and specificities herein were built from a subsampled dataset, limiting the chemical space covered. Nevertheless, identifying compounds associated with the harmful property of oxidative stress is mandatory for a model to be selected. Therefore, the MCC was adopted as the selection criterion favoring models with balanced sensitivities and specificities over models with excellent specificity despite low sensitivity. As a result, only models with MCC higher than 0.20 were considered.

To validate the models’ predictions, a prospective experimental validation was devised. The Enamine HTS collection was selected for its diversity and synthetic accessibility. The collection contained 1.8 million compounds, of which 160 were selected based on model predictions for experimental validation. Due to practical considerations, the main challenge was to make the selection from such a large number of compounds. Several strategies were proposed, one of them consisting of each academic partner individually providing a list of compounds to be tested based on their own filtering criteria. Although such an approach is appealing to analyze differences among models and their resulting selections, it was commonly decided that the average ensemble of predictions would be used as its performance on the holdout test set was one of the highest. Furthermore, to limit the number of chemicals to test, a selection procedure was adopted, factoring in the chemical space and the similarity with respect to the training set on top of the predicted activities. Consequently, the prospective validation not only evaluated the models through metrics of performance but also provided insights into the importance of the aforementioned aspects. Once the prospective dataset was filtered, projected onto the clustered reference chemical space, and labeled by similarity with respect to the training set, the compounds for which our models were more confident were selected. Filters on the concordance of predictions and on the applicability domains were tuned, due to the different distributions of compounds per cluster and per similarity and activity subclasses. This tuning allowed for the selection of compounds having the maximum agreement among models yet populating the similarity and activity clusters best while fitting the allocated budget.

Experimental results highlighted the consistency of model performances between the holdout test set and the prospective validation set, as exemplified by the value of 0.06 for both the average and the standard deviation of absolute differences of MCC. However, because of the prioritization of models with balanced sensitivities and specificities, the results showed an increase in false positives, with a reduction in the average and standard deviation of absolute differences between sensitivities of the holdout test and prospective validation sets of 0.04 and 0.13, respectively. This bias was reflected in lower average accuracies of 0.70 and 0.61 for the holdout test and prospective validation sets, respectively. However, on average, models successfully predicted 15 active compounds out of 19, evincing the usefulness of models to prioritize compounds for further analysis. Additionally, the experimental design provided insights into how models face complex endpoints in terms of chemical space, Log *P*, and similarity with respect to the training series. Models generalized the relationship between low Log *P* (Log *P* < 2.5) and compounds’ inactivity but failed to identify the few active compounds (3) present in this interval. On the other hand, models were unable to clearly differentiate active and inactive compounds when Log *P* was higher, leading to a high number of false positives. Interestingly, performance was better for dissimilar selections, stressing model generalizability.

Another important outcome of this study is that ensemble methods returned better performance than single models before model selection: the consensus model derived from the 11 selected models showed suboptimal performance. The integration of multiple modeling strategies and the application of a weight-of-evidence approach is particularly suited when the individual models have been developed using different techniques, have ADs differently defined, and show different behaviors based on the structural and activity profile of predicted chemicals.^59−61^ In this regard, the integrated method can compensate for and correct for the limitations of individual techniques, can cover greater chemical space, and increases confidence in the final toxicological prediction.

As a result, we recommend the use of consensus models derived from weak learners, as exemplified by model MN 1’s consistency in ranking in the top-performing models in all Log *P* intervals and almost all clusters of the reference chemical space.

In silico models developed herein can be used to provide information regarding the toxicity of chemicals and help to prioritize certain chemicals for further testing and give an indication to better plan targeted follow-up in vitro experiments. Overall, indications given by in silico methods were confirmed by the subsequent in vitro testing, confirming the suitability of these models as effecting top-tier methods within integrated testing strategies (ITS). Additionally, these models could be used in the active learning-based creation of a predictive modeling compound set for oxidative stress predictions.

The real-life applicability of the models has been one of the main focuses during the development and validation of this work. In particular, great attention has been put into the selection of chemicals for prospective validation. Indeed, the reference database was a combination of heterogeneous sources of chemicals (i.e., COSMOS, DrugBank, and ECHA6) with the explicit aim to validate the predictivity of models on a broad range of substances (e.g., drugs, cosmetics, nutraceuticals, toxic industrial chemicals) that can be released in the environment and can activate the oxidative stress pathway and hence cause toxicity.

## Conclusions

In this work, an NRF2 activity reporter was used to measure oxidative stress pathway activation in HepG2 cells. A large series of 2230 drug and drug-like compounds were classified as positive or negative depending on the cellular response and distributed among different modeling groups for building SAR classifier models. A selection of models was used to prospectively predict oxidative stress induced by a set of diverse compounds, which were then tested in vitro for validation. The setup presented here validated the models’ performance across the similarity and lipophilicity landscape. Additionally, failure modes of individual models were investigated and characterized based on training series. This work exemplifies the challenges of explaining machine learning model-based decisions in the context of AOP activation.

## Data Availability

The data used in this study are available in the Supporting Information.

## Supporting Information Available

Additional file 1: Details on the models’ performance and on the molecules presented (PDF)
Additional file 2: Aggregated training, holdout test, and validation sets (ZIP)
Additional file 3: SMILES of molecules of Table 4 (TXT)

## Abbreviations

AOP: adverse outcome pathway
BAC: bacterial artificial chromosome
BRF: balanced random forest
BS: bioactivity spectra
CDDO-Me: bardoxolone methyl (methyl-2-cyano 3,12-dioxooleano-1,9-dien-28-oate)
CLP: classification, labeling, and packaging regulation
CP: conformal prediction
DILI: drug-induced liver injury
DNN: deep neural networks
DT: decision tree
ECHA: European Chemicals Agency
FCFP_4: functional connectivity fingerprints with radius 2
FCFP_6: functional connectivity fingerprints with radius 3
FN: false negative
GBT: gradient-boosted tree
GFP: green fluorescent protein
ITS: integrated testing strategies
kNN: *k*-nearest neighbors
LR: logistic regression
MCC: Matthews correlation coefficient
MCP: Mondrian conformal prediction
NB: naive Bayes
NRF2: nuclear factor erythroid 2-related factor 2
PC: principal component
PHH: primary human hepatocytes
PhysChem: physicochemical descriptors
PLSR: partial least-squares regression
SAR: structure–activity relationships
RF: random forest
ROS: reactive oxygen species
Srxn1: sulfiredoxin
Srxn1-GFP: GFP-tagged NRF2-regulated sulfiredoxin reporter
SVM: support vector machine
Tc: Tanimoto coefficient/similarity
