# Supplementary material for: Collaborative SAR Modeling and Prospective In Vitro Validation of Oxidative Stress Activation in Human HepG2 Cells
Source: J Chem Inf Model. 2023 Aug 24;63(17):5433–45. doi: 10.1021/acs.jcim.3c00220 (PMC10498489; doi:10.1021/acs.jcim.3c00220)
Supplement: Supplementary file 1 — ci3c00220_si_001.pdf [file ci3c00220_si_001.pdf]

Supporting Information - Collaborative SAR modelling and prospective in vitro validation of oxidative stress activation in human HepG2 cells

Olivier J. M. Béquignon,[†,#] Jose C. Gómez-Tamayo,[‡,¶,#] Eelke B. Lenselink,[†,§] Steven Wink,[†] Steven Hiemstra,[†] Chi Chung Lam,[†] Domenico Gadaleta,[‖] Alessandra Roncaglioni,[‖] Ulf Norinder,[⊥] Bob van de Water,[†] Manuel Pastor,[‡] and Gerard J. P. van Westen*,[†]

†*Leiden Academic Centre for Drug Research, Leiden University, Wassenaarseweg 76, 2333 AL Leiden, The Netherlands*

‡*Research Programme on Biomedical Informatics (GRIB), Department of Medicine and Life Sciences, Universitat Pompeu Fabra, Hospital del Mar Medical Research Institute, Carrer del Dr. Aiguader 88, 08002 Barcelona, Spain*

¶*Current address: Computational Chemistry, Janssen Research & Development, Turnhoutseweg 30, Beerse B-2340, Belgium*

§*Current address: Galapagos NV, Generaal De Wittelaan L11 A3, 2800, Mechelen, Belgium*

‖*Laboratory of Environmental Chemistry and Toxicology, Department of Environmental Health Sciences, IRCCS - Istituto di Ricerche Farmacologiche Mario Negri, Via la Masa 19, 20156 Milano, Italy*

⊥*MTM Research Centre, School of Science and Technology, Örebro University, SE-70182 Örebro, Sweden*

#*O.J.M.B. and J.C.G.T. contributed equally to this paper*

E-mail: gerard@lacdr.leidenuniv.nl

Table S1: Model performances on the hold-out set.

model	actives:inactives	MCC	ACC	BACC	SN	SP	PPV	NPV	F1	TP	TN	FP	FN	AD
perfect	98:573	1.000	1.000	1.000	1.000	1.000	1.000	1.000	1.000	98	573	0	0	1.000
MN 1	48:302	0.439	0.837	0.757	0.646	0.868	0.437	0.939	0.521	31	262	40	17	0.522
Swetox 4	72:460	0.309	0.718	0.714	0.708	0.720	0.283	0.940	0.405	51	331	129	21	0.793
UL 10	98:573	0.305	0.793	0.676	0.510	0.841	0.355	0.909	0.418	50	482	91	48	1.000
Ensemble mean	98:573	0.293	0.800	0.663	0.469	0.857	0.359	0.904	0.407	46	491	82	52	1.000
Ensemble median	98:573	0.292	0.806	0.658	0.449	0.867	0.367	0.902	0.404	44	497	76	54	1.000
Swetox 2	71:441	0.286	0.725	0.692	0.648	0.737	0.284	0.929	0.395	46	325	116	25	0.763
UL 8	98:573	0.285	0.779	0.668	0.510	0.826	0.333	0.908	0.403	50	473	100	48	1.000
Swetox 3	84:527	0.265	0.696	0.683	0.667	0.700	0.262	0.930	0.376	56	369	158	28	0.911
UL 5	98:573	0.262	0.677	0.680	0.684	0.675	0.265	0.926	0.382	67	387	186	31	1.000
UPF 1	51:406	0.260	0.710	0.675	0.630	0.720	-	-	-	-	-	-	-	0.680
UPF 2	-	0.260	0.660	0.660	0.710	0.610	-	-	-	-	-	-	-	0.670
MN 8	98:571	0.254	0.786	0.642	0.439	0.846	0.328	0.898	0.376	43	483	88	55	0.997
UPF 3	-	0.250	0.660	0.680	0.700	0.660	-	-	-	-	-	-	-	0.710
UL 6	98:573	0.249	0.791	0.637	0.418	0.855	0.331	0.896	0.369	41	490	83	57	1.000
UPF 4	46:458	0.240	0.670	0.670	0.670	0.670	-	-	-	-	-	-	-	0.750
UL 3	98:573	0.239	0.836	0.600	0.265	0.934	0.406	0.881	0.321	26	535	38	72	1.000
UL 11	98:573	0.238	0.633	0.667	0.714	0.620	0.243	0.927	0.363	70	355	218	28	1.000
MN 4	98:573	0.231	0.692	0.654	0.602	0.707	0.260	0.912	0.363	59	405	168	39	1.000
MN 7	98:573	0.220	0.714	0.642	0.541	0.744	0.265	0.905	0.356	53	426	147	45	1.000
UL 13	98:573	0.219	0.847	0.576	0.194	0.958	0.442	0.874	0.270	19	549	24	79	1.000
UPF 5	66:430	0.210	0.700	0.645	0.570	0.720	-	-	-	-	-	-	-	0.740
Swetox 1	84:515	0.209	0.685	0.642	0.583	0.701	0.241	0.912	0.342	49	361	154	35	0.893
UL 12	98:573	0.206	0.808	0.600	0.306	0.894	0.330	0.883	0.318	30	512	61	68	1.000
MN 5	97:564	0.199	0.729	0.624	0.474	0.773	0.264	0.895	0.340	46	436	128	51	0.985
MN 6	98:573	0.184	0.638	0.627	0.612	0.642	0.226	0.906	0.331	60	368	205	38	1.000
UL 2	98:573	0.170	0.774	0.593	0.337	0.848	0.275	0.882	0.303	33	486	87	65	1.000
UL 1	98:573	0.170	0.623	0.619	0.612	0.625	0.218	0.904	0.322	60	358	215	38	1.000
MN 2	89:525	0.159	0.546	0.613	0.708	0.518	0.199	0.913	0.311	63	272	253	26	0.915
UL 7	98:573	0.145	0.818	0.559	0.194	0.925	0.307	0.870	0.238	19	530	43	79	1.000
UL 9	98:573	0.132	0.857	0.514	0.031	0.998	0.750	0.858	0.059	3	572	1	95	1.000
MN 3	98:573	0.129	0.604	0.590	0.571	0.609	0.200	0.893	0.296	56	349	224	42	1.000
UL 4	98:573	0.107	0.712	0.565	0.357	0.773	0.212	0.876	0.266	35	443	130	63	1.000
UL 14	98:573	0.050	0.520	0.535	0.550	0.520	-	-	-	-	-	-	-	1.000

Models are sorted by MCC. actives:inactives, MCC, ACC, BACC, SN, SP, PPV, NPV, F1, TP, TN, FP, FN and AD stand for number of active and inactive compounds falling within each model's applicability domain and for which predictions were obtained, Matthews correlation coefficient, accuracy, balanced accuracy, sensitivity, specificity, positive predicted value, negative predicted value, F1-score, number of true positives, true negatives, false positives, false negatives and coverage of the applicability domain respectively.

Table S2: Detailed information of compounds shown in Table 4

compound structure	name	ID	SMILES
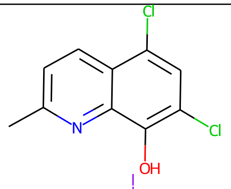	clorquinaldol	euspeclibcmpd1014	`Cc1ccc2c(Cl)cc(Cl)c(O)c2n1`
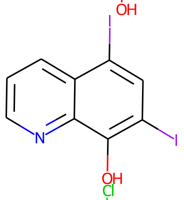	iodoquinol	euspeclibcmpd2015	`Oc1c(I)cc(I)c2cccnc12`
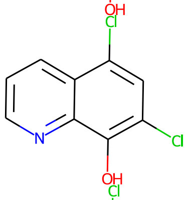	chloroxine	euspeclibcmpd537	`Oc1c(Cl)cc(Cl)c2cccnc12`
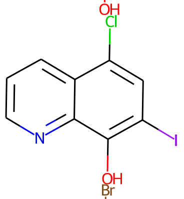	clioquinol	euspeclibcmpd705	`Oc1c(I)cc(Cl)c2cccnc12`
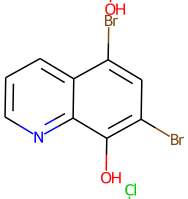	broxyquinoline	euspeclibcmpd1032	`Oc1c(Br)cc(Br)c2cccnc12`
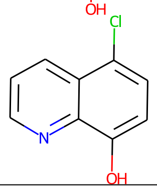	cloxyquin	euspeclibcmpd72	`Oc1ccc(Cl)c2cccnc12`

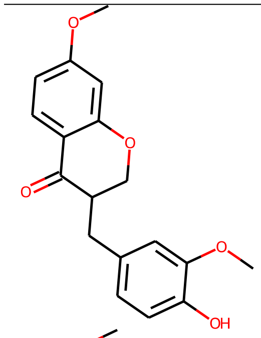	deoxysappanone B 7,3'-dimethyl ether	euspeclibcmpd1837	`COc1ccc2c(c1)OCC(Cc1ccc(O)c(OC)c1)C2=O`
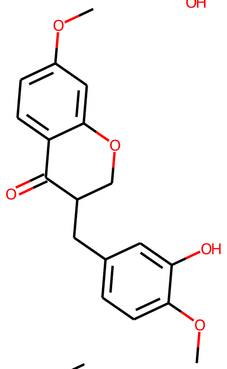	deoxysappanone B 7,4'-dimethyl ether	euspeclibcmpd1628	`COc1ccc2c(c1)OCC(Cc1ccc(OC)c(O)c1)C2=O`
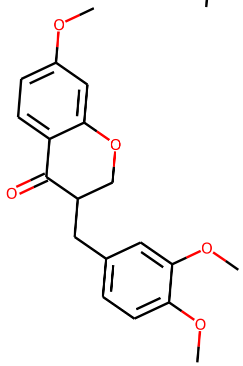	deoxysappanone B trimethyl ether	euspeclibcmpd1645	`COc1ccc2c(c1)OCC(Cc1ccc(OC)c(OC)c1)C2=O`

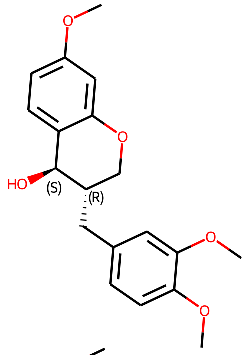	3-deshydroxy-sappanol trimethyl ether	euspeclibcmpd2183	`COc1ccc2c(c1)OC[C@@H](Cc1ccc(OC)c(OC)c1)[C@@H]2O`
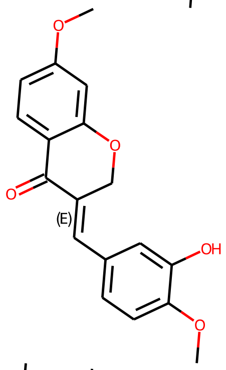	sappanone A dimethyl ether	euspeclibcmpd1638	`COc1ccc2c(c1)OC/C(=C1ccc(OC)c(O)c1)C2=O`
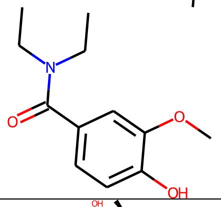	ethamivan	euspeclibcmpd904	`CCN(CC)C(=O)c1ccc(O)c(OC)c1`
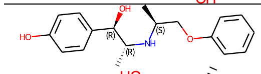	isoxsuprine	euspeclibcmpd167	`C[C@@H](COc1ccccc1)N[C@H](C)[C@H](O)c1ccc(O)cc1`
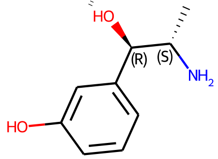	metaraminol	euspeclibcmpd554	`C[C@H](N)[C@H](O)c1cccc(O)c1`

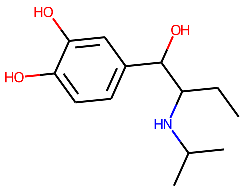	isoetharine	euspeclibcmpd894	`CCC(NC(C)C)C(O)c1ccc(O)c(O)c1`
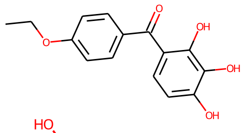	2,3,4-trihydroxy-4’-ethoxy benzophenone	euspeclibcmpd1369	`CCOc1ccc(C(=O)c2ccc(O)c(O)c2O)cc1`
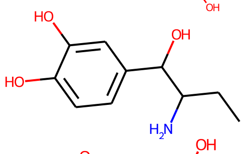	ethylnorepinephrine	euspeclibcmpd646	`CCC(N)C(O)c1ccc(O)c(O)c1`
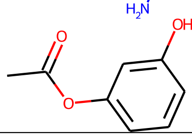	resorcinol monoacetate	euspeclibcmpd584	`CC(=O)Oc1cccc(O)c1`

Table S3: Model performance on the validation test set per cluster of the reference chemical space

cluster	model	actives:inactives	MCC	ACC	BACC	SN	SP	PPV	NPV	F1	TP	TN	FP	FN
0	UL 1	03:18	0.550	0.900	0.670	0.330	1.000	1.000	0.900	0.500	1	18	0	2
	MN 1	03:18	0.500	0.860	0.780	0.670	0.890	0.500	0.940	0.570	2	16	2	1
	MN 4	03:18	0.290	0.710	0.690	0.670	0.720	0.290	0.930	0.400	2	13	5	1
	Swetox 1	03:18	0.290	0.710	0.690	0.670	0.720	0.290	0.930	0.400	2	13	5	1
	Swetox 3	03:18	0.290	0.710	0.690	0.670	0.720	0.290	0.930	0.400	2	13	5	1
	UL 11	03:18	0.290	0.710	0.690	0.670	0.720	0.290	0.930	0.400	2	13	5	1
	UL 5	03:18	0.290	0.710	0.690	0.670	0.720	0.290	0.930	0.400	2	13	5	1
	UL 8	03:18	0.290	0.710	0.690	0.670	0.720	0.290	0.930	0.400	2	13	5	1
	UPF 3	03:18	0.290	0.710	0.690	0.670	0.720	0.290	0.930	0.400	2	13	5	1
	Consensus	03:18	0.290	0.710	0.690	0.670	0.720	0.290	0.930	0.400	2	13	5	1
	UPF 1	02:16	0.240	0.780	0.660	0.500	0.810	0.250	0.930	0.330	1	13	3	1
	UPF 4	02:18	0.150	0.700	0.610	0.500	0.720	0.170	0.930	0.250	1	13	5	1
1	UPF 4	05:37	0.300	0.520	0.730	1.000	0.460	0.200	1.000	0.330	5	17	20	0
	UPF 1	08:39	0.280	0.550	0.680	0.880	0.490	0.260	0.950	0.400	7	19	20	1
	MN 1	08:44	0.210	0.580	0.650	0.750	0.550	0.230	0.920	0.350	6	24	20	2
	MN 4	08:44	0.210	0.480	0.640	0.880	0.410	0.210	0.950	0.340	7	18	26	1
	UL 11	08:44	0.210	0.480	0.640	0.880	0.410	0.210	0.950	0.340	7	18	26	1
	Swetox 3	08:44	0.200	0.460	0.630	0.880	0.390	0.210	0.940	0.330	7	17	27	1
	UL 8	08:44	0.200	0.460	0.630	0.880	0.390	0.210	0.940	0.330	7	17	27	1
	Consensus	08:44	0.200	0.460	0.630	0.880	0.390	0.210	0.940	0.330	7	17	27	1
	UPF 3	08:44	0.180	0.540	0.630	0.750	0.500	0.210	0.920	0.330	6	22	22	2
	UL 1	08:44	0.180	0.690	0.610	0.500	0.730	0.250	0.890	0.330	4	32	12	4
	UL 5	08:44	0.170	0.420	0.610	0.880	0.340	0.190	0.940	0.320	7	15	29	1
	Swetox 1	08:42	0.120	0.460	0.580	0.750	0.400	0.190	0.890	0.310	6	17	25	2
2	MN 1	00:06	0.000	1.000	-	0.000	1.000	0.000	1.000	0.000	0	6	0	0
	Swetox 1	00:06	0.000	1.000	-	0.000	1.000	0.000	1.000	0.000	0	6	0	0
	Swetox 3	00:06	0.000	1.000	-	0.000	1.000	0.000	1.000	0.000	0	6	0	0
	UL 1	00:06	0.000	1.000	-	0.000	1.000	0.000	1.000	0.000	0	6	0	0
	UL 11	00:06	0.000	1.000	-	0.000	1.000	0.000	1.000	0.000	0	6	0	0
	UL 5	00:06	0.000	1.000	-	0.000	1.000	0.000	1.000	0.000	0	6	0	0
	UL 8	00:06	0.000	1.000	-	0.000	1.000	0.000	1.000	0.000	0	6	0	0
	UPF 1	00:06	0.000	1.000	-	0.000	1.000	0.000	1.000	0.000	0	6	0	0
	UPF 3	00:06	0.000	1.000	-	0.000	1.000	0.000	1.000	0.000	0	6	0	0
	UPF 4	00:06	0.000	1.000	-	0.000	1.000	0.000	1.000	0.000	0	6	0	0
	Consensus	00:06	0.000	1.000	-	0.000	1.000	0.000	1.000	0.000	0	6	0	0
	MN 4	00:06	0.000	0.830	-	0.000	0.830	0.000	1.000	0.000	0	5	1	0
3	MN 1	03:16	0.540	0.890	0.670	0.330	1.000	1.000	0.890	0.500	1	16	0	2

3	UPF 1	03:15	0.540	0.890	0.670	0.330	1.000	1.000	0.880	0.500	1	15	0	2
	UPF 3	03:16	0.400	0.790	0.740	0.670	0.810	0.400	0.930	0.500	2	13	3	1
	UL 11	03:16	0.320	0.840	0.640	0.330	0.940	0.500	0.880	0.400	1	15	1	2
	UL 5	03:16	0.320	0.840	0.640	0.330	0.940	0.500	0.880	0.400	1	15	1	2
	UPF 4	03:16	0.320	0.840	0.640	0.330	0.940	0.500	0.880	0.400	1	15	1	2
	Consensus	03:16	0.320	0.840	0.640	0.330	0.940	0.500	0.880	0.400	1	15	1	2
	Swetox 3	03:14	0.310	0.820	0.630	0.330	0.930	0.500	0.870	0.400	1	13	1	2
	MN 4	03:16	0.210	0.790	0.600	0.330	0.880	0.330	0.880	0.330	1	14	2	2
	Swetox 1	03:16	0.210	0.790	0.600	0.330	0.880	0.330	0.880	0.330	1	14	2	2
	UL 1	03:16	-0.100	0.790	0.470	0.000	0.940	0.000	0.830	0.000	0	15	1	3
	UL 8	03:16	-0.100	0.790	0.470	0.000	0.940	0.000	0.830	0.000	0	15	1	3
4	UPF 1	05:49	0.300	0.560	0.760	1.000	0.510	0.170	1.000	0.290	5	25	24	0
	UL 5	05:56	0.280	0.540	0.750	1.000	0.500	0.150	1.000	0.260	5	28	28	0
	Swetox 1	04:49	0.260	0.530	0.740	1.000	0.490	0.140	1.000	0.240	4	24	25	0
	UPF 4	05:49	0.240	0.440	0.690	1.000	0.390	0.140	1.000	0.250	5	19	30	0
	MN 4	05:56	0.230	0.460	0.710	1.000	0.410	0.130	1.000	0.230	5	23	33	0
	UL 11	05:56	0.230	0.460	0.710	1.000	0.410	0.130	1.000	0.230	5	23	33	0
	UL 8	05:56	0.230	0.460	0.710	1.000	0.410	0.130	1.000	0.230	5	23	33	0
	Consensus	05:56	0.230	0.460	0.710	1.000	0.410	0.130	1.000	0.230	5	23	33	0
	Swetox 3	05:56	0.220	0.430	0.690	1.000	0.380	0.130	1.000	0.220	5	21	35	0
	UPF 3	05:56	0.110	0.430	0.600	0.800	0.390	0.110	0.960	0.190	4	22	34	1
	MN 1	05:56	0.080	0.700	0.570	0.400	0.730	0.120	0.930	0.180	2	41	15	3
	UL 1	05:56	0.060	0.820	0.540	0.200	0.880	0.130	0.920	0.150	1	49	7	4
5	MN 1	00:01	0.000	1.000	-	0.000	1.000	0.000	1.000	0.000	0	1	0	0
	MN 4	00:01	0.000	1.000	-	0.000	1.000	0.000	1.000	0.000	0	1	0	0
	Swetox 1	00:01	0.000	1.000	-	0.000	1.000	0.000	1.000	0.000	0	1	0	0
	Swetox 3	00:01	0.000	1.000	-	0.000	1.000	0.000	1.000	0.000	0	1	0	0
	UL 1	00:01	0.000	1.000	-	0.000	1.000	0.000	1.000	0.000	0	1	0	0
	UL 11	00:011	0.000	1.000	-	0.000	1.000	0.000	1.000	0.000	0	1	0	0
	UL 5	00:01	0.000	1.000	-	0.000	1.000	0.000	1.000	0.000	0	1	0	0
	UL 8	00:01	0.000	1.000	-	0.000	1.000	0.000	1.000	0.000	0	1	0	0
	UPF 1	00:01	0.000	1.000	-	0.000	1.000	0.000	1.000	0.000	0	1	0	0
	UPF 3	00:01	0.000	1.000	-	0.000	1.000	0.000	1.000	0.000	0	1	0	0
	UPF 4	00:01	0.000	1.000	-	0.000	1.000	0.000	1.000	0.000	0	1	0	0
	UPF 4	00:01	0.000	1.000	-	0.000	1.000	0.000	1.000	0.000	0	1	0	0

Models are sorted by MCC for each cluster of the chemical space defined in Figure 2. actives:inactives, MCC, ACC, BACC, SN, SP, PPV, NPV, F1, TP, TN, FP and FN stand for the number of active and inactive compounds falling within each model's applicability domain and for which predictions were obtained, Matthews correlation coefficient, accuracy, balanced accuracy, sensitivity, specificity, positive predicted value, negative predicted value, F1-score, number of true positives, true negatives, false positives and false negatives respectively.

Table S4: Model performance on the validation test set per LogP interval

logp class	model	actives:inactives	MCC	ACC	BACC	SN	SP	PPV	NPV	F1	TP	TN	FP	FN
LogP < 0	UPF 3	02:11	0.270	0.770	0.660	0.500	0.820	0.330	0.900	0.400	1	9	2	1
	UPF 4	02:11	0.000	0.850	0.500	0.000	1.000	0.000	0.850	0.000	0	11	0	2
	MN 1	02:11	0.000	0.850	0.500	0.000	1.000	0.000	0.850	0.000	0	11	0	2
	Swetox 1	02:11	0.000	0.850	0.500	0.000	1.000	0.000	0.850	0.000	0	11	0	2
	MN 4	02:11	0.000	0.850	0.500	0.000	1.000	0.000	0.850	0.000	0	11	0	2
	Swetox 3	02:11	0.000	0.850	0.500	0.000	1.000	0.000	0.850	0.000	0	11	0	2
	UL 1	02:11	0.000	0.850	0.500	0.000	1.000	0.000	0.850	0.000	0	11	0	2
	UL 5	02:11	0.000	0.850	0.500	0.000	1.000	0.000	0.850	0.000	0	11	0	2
	UL 8	02:11	0.000	0.850	0.500	0.000	1.000	0.000	0.850	0.000	0	11	0	2
	UL 11	02:11	0.000	0.850	0.500	0.000	1.000	0.000	0.850	0.000	0	11	0	2
	Consensus	02:11	0.000	0.850	0.500	0.000	1.000	0.000	0.850	0.000	0	11	0	2
	UPF 1	02:09	0.000	0.820	0.500	0.000	1.000	0.000	0.820	0.000	0	9	0	2
0 < LogP ≤ 2.5	MN 1	01:49	0.000	0.980	0.500	0.000	1.000	0.000	0.980	0.000	0	49	0	1
	UL 1	01:49	0.000	0.980	0.500	0.000	1.000	0.000	0.980	0.000	0	49	0	1
	UL 8	01:49	0.000	0.980	0.500	0.000	1.000	0.000	0.980	0.000	0	49	0	1
	UL 11	01:49	0.000	0.980	0.500	0.000	1.000	0.000	0.980	0.000	0	49	0	1
	Consensus	01:49	0.000	0.980	0.500	0.000	1.000	0.000	0.980	0.000	0	49	0	1
	UPF 4	01:48	0.000	0.980	0.500	0.000	1.000	0.000	0.980	0.000	0	48	0	1
	Swetox 3	01:48	0.000	0.980	0.500	0.000	1.000	0.000	0.980	0.000	0	48	0	1
	Swetox 1	01:49	-0.020	0.960	0.490	0.000	0.980	0.000	0.980	0.000	0	48	1	1
	UPF 3	01:49	-0.020	0.960	0.490	0.000	0.980	0.000	0.980	0.000	0	48	1	1
	UPF 1	01:48	-0.020	0.960	0.490	0.000	0.980	0.000	0.980	0.000	0	47	1	1
	MN 4	01:49	-0.030	0.940	0.480	0.000	0.960	0.000	0.980	0.000	0	47	2	1
	UL 5	01:49	-0.030	0.940	0.480	0.000	0.960	0.000	0.980	0.000	0	47	2	1
2.5 < LogP ≤ 5	UPF 1	14:62	0.240	0.460	0.640	0.930	0.350	0.250	0.960	0.390	13	22	40	1
	UPF 4	11:55	0.210	0.350	0.610	1.000	0.220	0.200	1.000	0.340	11	12	43	0
	MN 1	15:68	0.190	0.590	0.620	0.670	0.570	0.260	0.890	0.370	10	39	29	5
	UL 5	15:68	0.180	0.390	0.600	0.930	0.260	0.220	0.950	0.350	14	18	50	1
	MN 4	15:68	0.160	0.360	0.580	0.930	0.240	0.210	0.940	0.350	14	16	52	1
	UL 11	15:68	0.160	0.360	0.580	0.930	0.240	0.210	0.940	0.350	14	16	52	1
	Consensus	15:68	0.150	0.350	0.577	0.930	0.220	0.210	0.940	0.340	14	15	53	1
	UL 1	15:68	0.140	0.700	0.580	0.400	0.760	0.270	0.850	0.320	6	52	16	9
	Swetox 1	15:60	0.120	0.390	0.570	0.870	0.270	0.230	0.890	0.360	13	16	44	2
	Swetox 3	11:67	0.120	0.320	0.560	0.930	0.180	0.200	0.920	0.330	14	12	55	1
	UL 8	15:68	0.080	0.340	0.540	0.870	0.220	0.200	0.880	0.320	13	15	53	2
	UPF 3	15:68	0.080	0.390	0.550	0.800	0.290	0.200	0.870	0.320	12	20	48	3
5 < LogP	MN 1	01:13	0.210	0.430	0.690	1.000	0.380	0.110	1.000	0.200	1	5	8	0

5 < LogP	UPF 1	01:07	0.140	0.250	0.570	1.000	0.140	0.140	1.000	0.250	1	1	6	0
	UL 5	01:13	0.110	0.210	0.580	1.000	0.150	0.080	1.000	0.150	1	2	11	0
	UPF 4	01:13	0.000	0.070	0.500	1.000	0.000	0.070	0.000	0.130	1	0	13	0
	MN 4	01:13	0.000	0.070	0.500	1.000	0.000	0.070	0.000	0.130	1	0	13	0
	UPF 3	01:13	0.000	0.070	0.500	1.000	0.000	0.070	0.000	0.130	1	0	13	0
	Swetox 3	01:13	0.000	0.070	0.500	1.000	0.000	0.070	0.000	0.130	1	0	13	0
	UL 8	01:13	0.000	0.070	0.500	1.000	0.000	0.070	0.000	0.130	1	0	13	0
	UL 11	01:13	0.000	0.070	0.500	1.000	0.000	0.070	0.000	0.130	1	0	13	0
	Consensus	01:13	0.000	0.070	0.500	1.000	0.000	0.070	0.000	0.130	1	0	13	0
	Swetox 1	01:12	0.000	0.000	-	0.000	0.000	0.000	0.000	0.000	0	0	12	0
	UL 1	01:13	-0.180	0.640	0.350	0.000	0.690	0.000	0.900	0.000	0	9	4	1

Models are sorted by MCC for each LogP interval. actives:inactives, MCC, ACC, BACC, SN, SP, PPV, NPV, F1, TP, TN, FP and FN stand for the number of active and inactive compounds falling within each model's applicability domain and for which predictions were obtained, Matthews correlation coefficient, accuracy, balanced accuracy, sensitivity, specificity, positive predicted value, negative predicted value, F1-score, number of true positives, true negatives, false positives and false negatives respectively.

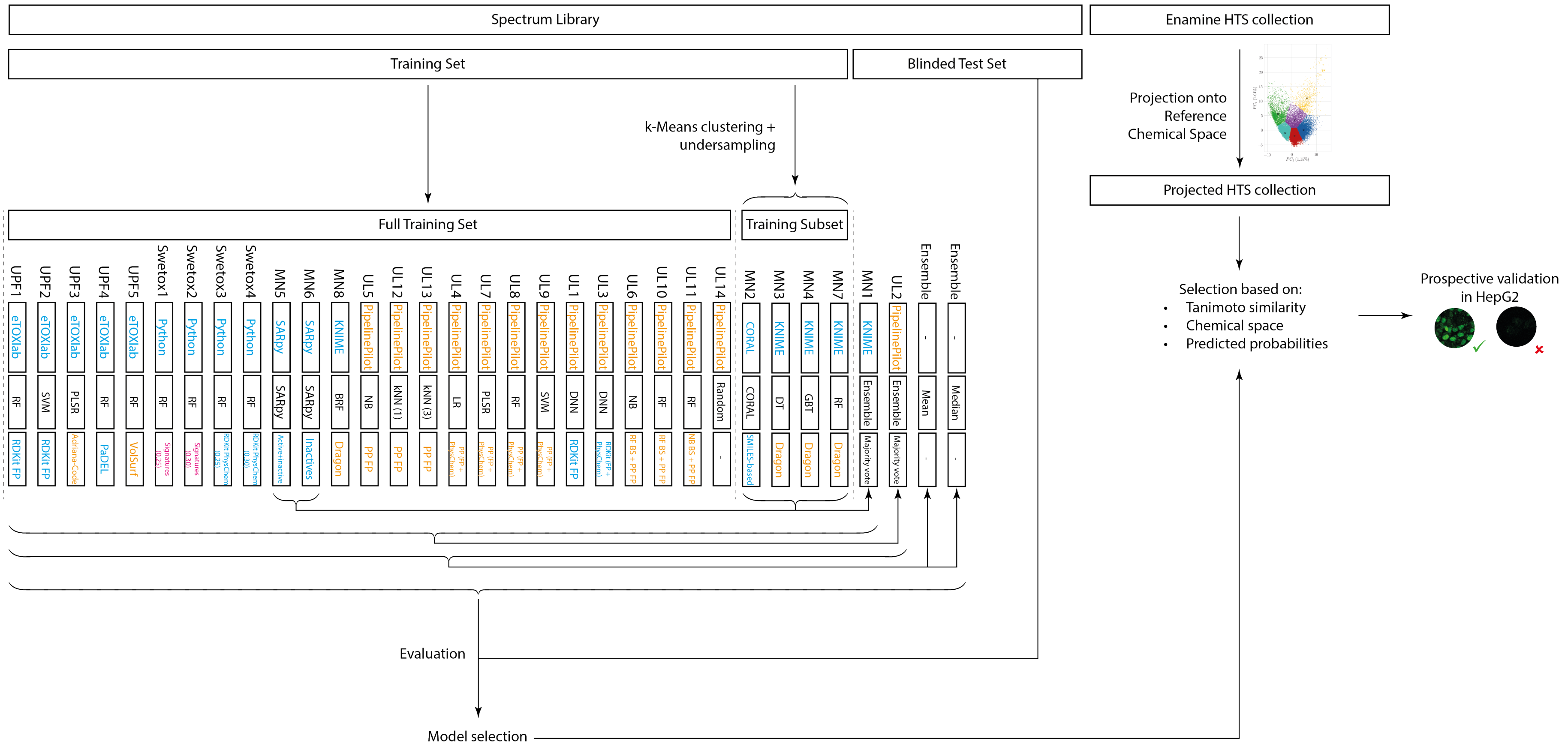

Figure S1: Overview of the methods applied to derive each machine learning model. Blue, orange, and pink annotations denote open-source, proprietary, and inaccessible software and descriptor types respectively.

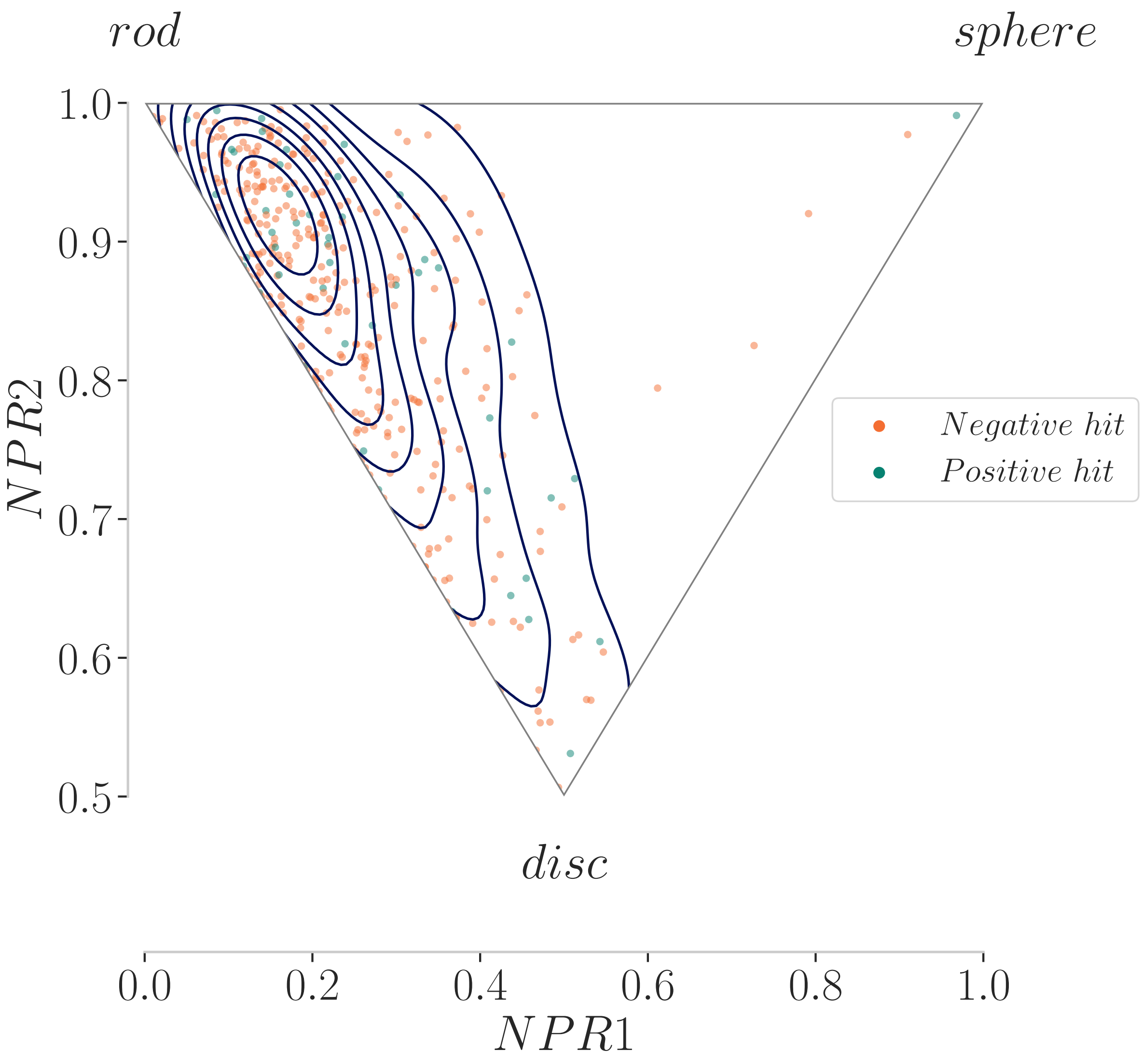

Figure S2: Normalized ratios of principal moments of inertia of molecules of the training and test set. Blue lines: kernel-density estimates.[1]

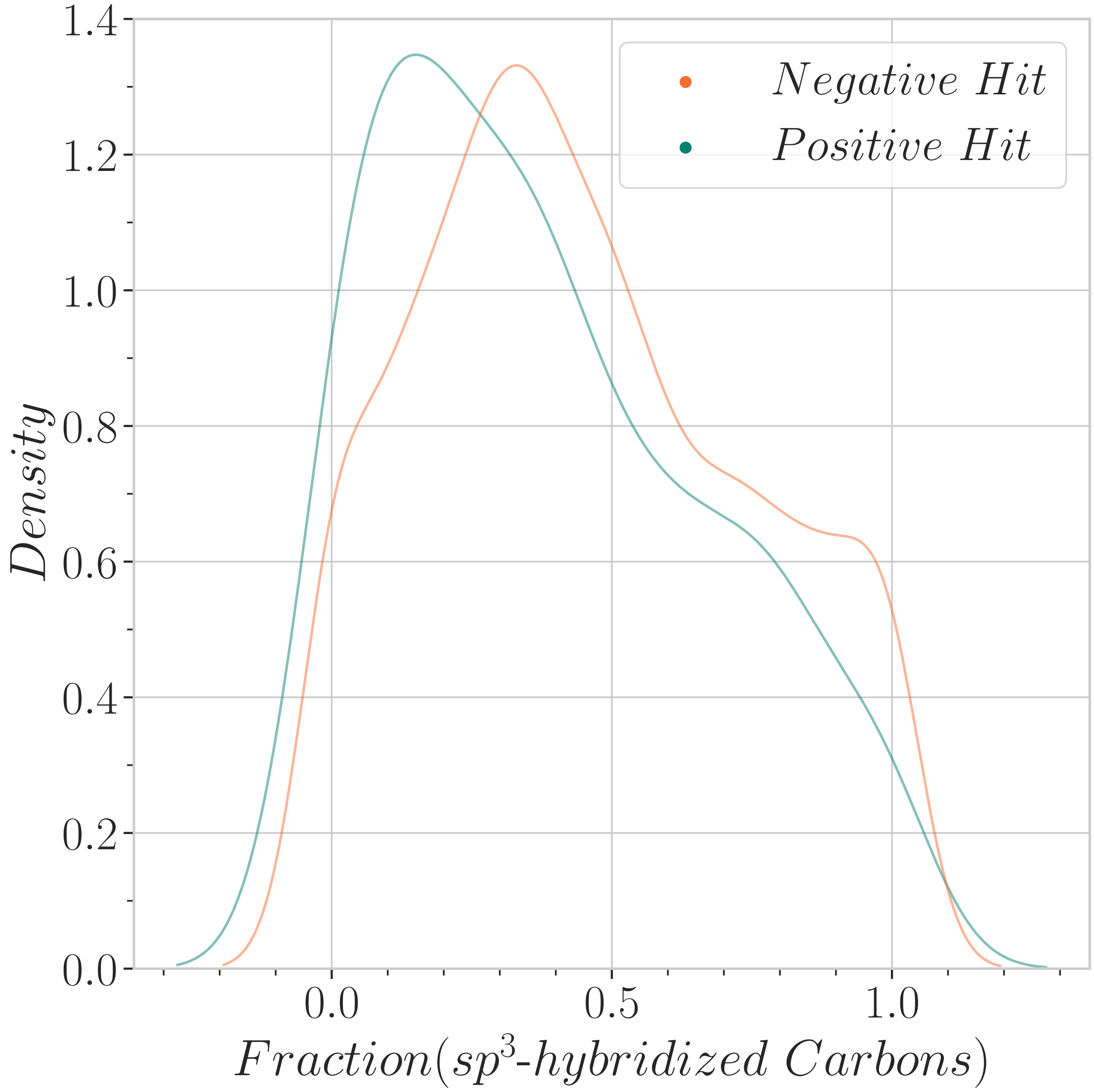

Figure S3: Kernel density estimate of the fraction of sp^3-hybridised molecules.

References

(1) Sauer, W. H. B.; Schwarz, M. K. Molecular Shape Diversity of Combinatorial Libraries: A Prerequisite for Broad Bioactivity. *J. Chem. Inf. Comput. Sci.* **2003**, *43*.
